# Low skeletal muscle mass is associated with increased hospital expenditure in patients undergoing cancer surgery of the alimentary tract

**DOI:** 10.1371/journal.pone.0186547

**Published:** 2017-10-31

**Authors:** Jeroen L. A. van Vugt, Stefan Buettner, Stef Levolger, Robert R. J. Coebergh van den Braak, Mustafa Suker, Marcia P. Gaspersz, Ron W. F. de Bruin, Cornelis Verhoef, Casper H. C. van Eijck, Niek Bossche, Bas Groot Koerkamp, Jan N. M. IJzermans

**Affiliations:** 1 Department of Surgery, Erasmus MC University Medical Centre, Rotterdam, the Netherlands; 2 Department of Control and Compliance, Erasmus MC University Medical Centre, Rotterdam, the Netherlands; 3 Department of Surgical Oncology, Erasmus MC Cancer Institute, Rotterdam, the Netherlands; Universidade de Mogi das Cruzes, BRAZIL

## Abstract

**Background:**

Low skeletal muscle mass is associated with poor postoperative outcomes in cancer patients. Furthermore, it is associated with increased healthcare costs in the United States. We investigated its effect on hospital expenditure in a Western-European healthcare system, with universal access.

**Methods:**

Skeletal muscle mass (assessed on CT) and costs were obtained for patients who underwent curative-intent abdominal cancer surgery. Low skeletal muscle mass was defined based on pre-established cut-offs. The relationship between low skeletal muscle mass and hospital costs was assessed using linear regression analysis and Mann-Whitney U-tests.

**Results:**

452 patients were included (median age 65, 61.5% males). Patients underwent surgery for colorectal cancer (38.9%), colorectal liver metastases (27.4%), primary liver tumours (23.2%), and pancreatic/periampullary cancer (10.4%). In total, 45.6% had sarcopenia. Median costs were €2,183 higher in patients with low compared with patients with high skeletal muscle mass (€17,144 versus €14,961; *P*<0.001). Hospital costs incrementally increased with lower sex-specific skeletal muscle mass quartiles (*P* = 0.029). After adjustment for confounders, low skeletal muscle mass was associated with a cost increase of €4,061 (*P* = 0.015).

**Conclusion:**

Low skeletal muscle mass was independently associated with increased hospital costs of about €4,000 per patient. Strategies to reduce skeletal muscle wasting could reduce hospital costs in an era of incremental healthcare costs and an increasingly ageing population.

## Introduction

Low skeletal muscle mass is a strong predictor of complications, reduced therapy effect, impaired survival in gastrointestinal and hepatopancreatobiliary cancer patients undergoing surgery [1–3], and dose-limiting chemotherapy toxicity [4–6]. In addition to clinical outcome, recent studies from the United States showed that low skeletal muscle mass is associated with increased healthcare costs [7–9].

However, the lower accessibility and higher uninsured rate of the American healthcare system, which is known to be greatly affected by income [10], may impair extrapolation to other healthcare systems with universal access [11–13]. Because European healthcare costs are increasingly rising, we aimed to assess the effect of low skeletal muscle mass on hospital costs of patients undergoing curative-intent surgery for abdominal cancer in a Western-European healthcare system.

## Materials and methods

### Patients and data acquisition

In this retrospective study, patients aged 18 years or older who underwent curative surgery for gastrointestinal or hepatopancreatobiliary cancers in our centre were identified from various databases (including colorectal carcinoma [14–16], hepatocellular carcinoma [17], colorectal liver metastases [18], perihilar [19] or intrahepatic [19] cholangiocarcinoma, and pancreatic or periampullary cancer [20]). Patients underwent surgery between 2005 and 2015. Only patients with a CT imaging within 90 days preoperatively were included. Demographics and patient characteristics were collected from electronic patient files. Preoperative physical status was assessed using the American Society of Anesthesiologists (ASA) score [21]. Overweight was defined as a body mass index (BMI) ≥ 25 kg/m^2^, according to the definition of the World Health Organization [22]. Postoperative complications were identified in the medical patient files and recorded. Severity of postoperative complications was scored according to the Clavien-Dindo classification [23]. Severe complications were defined as complications with grade 3a or higher. Postoperative mortality was defined as mortality during hospital stay or within 30 days after surgery. Length of hospital stay was calculated by counting admission days from the day of surgery. An hospital stay exceeding 7 days was considered a prolonged hospital stay. Patients were divided in two groups according to the extent of surgery: 1. Major surgery (i.e. the resection of at least two hepatic segments and a wedge resection or the resection of at least three hepatic segments, pancreatic surgery, and pelvic exenteration for locally advanced rectal cancer); 2. Minor surgery (i.e. one- or two hepatic segment resections, and colorectal resections). The study was approved by the Medical Ethical Committee of Erasmus MC University Medical Centre and a waiver for informed consent was granted.

### Skeletal muscle mass measurements

Skeletal muscle mass was measured on computed tomography (CT) examinations that were routinely performed as part of diagnostic or preoperative work-up, as previously described [18]. In short, the cross-sectional skeletal muscle area was measured at the level of the third lumbar vertebra on a slice on which both transversal processes were visible using a Hounsfield unit threshold of -30 to +150. The cross-sectional muscle area was then corrected for patients’ height squared, as is conventional for body composition measurements, resulting in the skeletal muscle index (SMI, cm^2^/m^2^). Patients were classified as having low skeletal muscle mass according to predefined cut-off values established by Martin and colleagues: males with body mass index (BMI) <25: <43 cm^2^/m^2^, males with BMI ≥25: <53 cm^2^/m^2^, females: <41 cm^2^/m^2^ [24]. Furthermore, patients were divided in sex-specific quartiles based on their skeletal muscle index. In patients who underwent induction or neoadjuvant chemo(radio)therapy, the CT after chemotherapy was used.

### Cost analyses

Costs were abstracted from the hospital’s electronic accounting system, which is based on activity based costing. All costs that were made during the index admission for surgery, including surgical costs and all costs in the postoperative period and independent of (medical) discipline, were included. Total costs were calculated by the sum of all unit cost-prices. These cost prices are covering the full cost prices. Financial data was limited to expenses within the index admission and did not include costs after discharge from the index admission or costs made outside our centre. Adjustment for inflation was performed by indexing all cost-prices to the year 2015 according to data of the Dutch Healthcare Authority. All financial data are reported in Euros (€).

### Statistical analyses

Categorical data are reported as counts with percentages, whereas continuous data are reported as median with interquartile range (IQR) or mean with standard deviation (SD), depending on their normality of distribution. Categorical data was compared using the chi-squared test. The Mann-Whitney-U and Kruskall-Wallis H tests was used to compare hospital costs between groups. A multivariable linear regression analysis was performed to investigate the independent association of the presence of low skeletal muscle mass with increased costs after correction for possible confounding and clinically relevant factors. Sex-specific skeletal muscle mass quartiles were compared using one-way ANOVA analysis. Continuous baseline and outcome characteristics were dichotomized, according to clinically relevant cut-off points, and subgroup analyses were performed to investigate the effect of low skeletal muscle mass on total hospital costs in various subgroups. Patients who died during hospital admission or within 30 days postoperative were excluded from the subgroup analyses based on length of hospital stay. Two-sided *P*-values <0.05 were considered statistically significant. All analyses were performed using SPSS for Windows (IBM Corp., Armonk, NY, USA), version 22.

## Results

### Patients

In total, 602 patients were identified. Of these patients, no preoperative CT was available in 83 patients (13.8%) and no costs were available for 18 (3.0%) patients. In 49 patients (8.1%) the CT was not performed within 90 days preoperatively. This resulted in 452 patients (75.1%) who formed the study cohort, of whom 278 (61.5%) were male and 174 (38.5%) female with a median age of 65 (IQR 58–71) years. Most patients underwent surgery for colorectal carcinoma (*N* = 176, 38.9%), followed by colorectal liver metastases (*N* = 124, 27.4%), hepatocellular carcinoma (*N* = 53, 11.7%), pancreatic or periampullary cancer (*N* = 47, 10.4%), intrahepatic cholangiocarcinoma (*N* = 32, 7.1%), and perihilar cholangiocarcinoma (*N* = 20, 4.4%). Baseline characteristics are summarized in Table 1.

**Table 1:** Baseline characteristics.

	All patients (*N* = 452)	Low skeletal muscle mass (*N* = 206)	Normal skeletal muscle mass (*N* = 246)	*P*-value
**Sex**				
Males	278 (61.5)	111 (53.9)	167 (67.9)	0.002
Females	174 (38.5)	95 (46.1)	79 (32.1)	
**Age (years)**	64.7 (57.8–71.4)	65.1 (58.3–73.0)	64.5 (57.2–70.3)	0.069
**BMI (kg/m^2^)**	25.2 (22.7–27.9)	25.1 (21.8–27.2)	25.2 (23.4–28.6)	0.008
**ASA classification**^**#**^				
1–2	334 (79.3)	154 (78.6)	180 (80.0)	0.718
3–4	87 (20.7)	42 (21.4)	45 (20.0)	
**Cancer diagnosis**				
Colorectal	176 (38.9)	75 (36.4)	101 (41.1)	<0.001
CRLM	124 (27.4)	38 (18.4)	86 (35.0)	
HCC	53 (11.7)	27 (13.1)	26 (10.6)	
Pancreatic/periampullary	47 (10.4)	34 (16.5)	13 (5.3)	
ICC	32 (7.1)	19 (9.2)	13 (5.3)	
PHC	20 (4.4)	13 (6.3)	7 (2.8)	

Abbreviations: BMI, Body Mass Index; ASA, American Society for Anesthesiologists (^#^ missing for 31 patients); CRLM, Colorectal Liver Metastases; HCC, Hepatocellular Carcinoma; ICC, Intrahepatic Cholangiocarcinoma; PHC, Perihilar Cholangiocarcinoma.

### The association between low skeletal muscle mass and treatment outcomes

Almost half of our cohort (45.6%, *N* = 206) had low skeletal muscle mass. Patients with low skeletal muscle mass experienced more postoperative complications compared with patients with normal skeletal muscle mass (55.1% versus 44.9%, *P* = 0.031), and showed an increased length of hospital stay (median 9 [IQR 7–14] versus 8 [IQR 6–12] days, *P* = 0.005). Non-significant differences were observed for severe postoperative complications and postoperative mortality (Table 2).

**Table 2:** Treatment outcomes.

	Low skeletal muscle mass (*N* = 206)	Normal skeletal muscle mass (*N* = 246)	*P*-value
**Any postoperative complication**	113 (55.1)	110 (44.9)	0.031
Any severe postoperative complication*	48 (23.4)	47 (19.2)	0.273
**Postoperative mortality**^#^	15 (7.3)	9 (3.7)	0.087
**Length of hospital stay (days)**^‡^	9 (7–14)	8 (6–12)	0.005

* Defined as Clavien-Dindo classification ≥ 3a

^#^ Defined as in-hospital or 30-day mortality

^‡^ Without patients who died in-hospital or within 30 days postoperatively

### Total hospital costs in patients with low and normal skeletal muscle mass

The median total hospital costs per patient were €16,021 (IQR 11,714–23,381). Total hospital costs significantly differed between cancer types, and were highest in patients operated on for perihilar cholangiocarcinoma and lowest in patients who underwent surgery for colorectal liver metastases (Table 3). Patients undergoing major surgery had significantly higher costs compared with patients undergoing minor surgery (€21,124 [IQR 14,618–29,884] versus €12,989 [IQR 10,029–16,736], p<0.001).

**Table 3:** Total hospital costs per cancer type.

Cancer type	Total hospital costs, € (IQR)	p-value
**Colorectal**	15,121 (11,718–19,945)	<0.001
**CRLM**	12,431 (8,721–14,679)	
**HCC**	22,396 (16,368–32,474)	
**Pancreatic/periampullary**	22,057 (18,509–25,445)	
**ICC**	30,130 (18,710–40,827)	
**PHC**	36,542 (28,122–53,126)	

Abbreviations: IQR, Interquartile Range; CRLM, Colorectal Liver Metastases, HCC, Hepatocellular Carcinoma; ICC, Intrahepatic Cholangiocarcinoma; PHC, Perihilar Cholangiocarcinoma.

**Fig 1:**
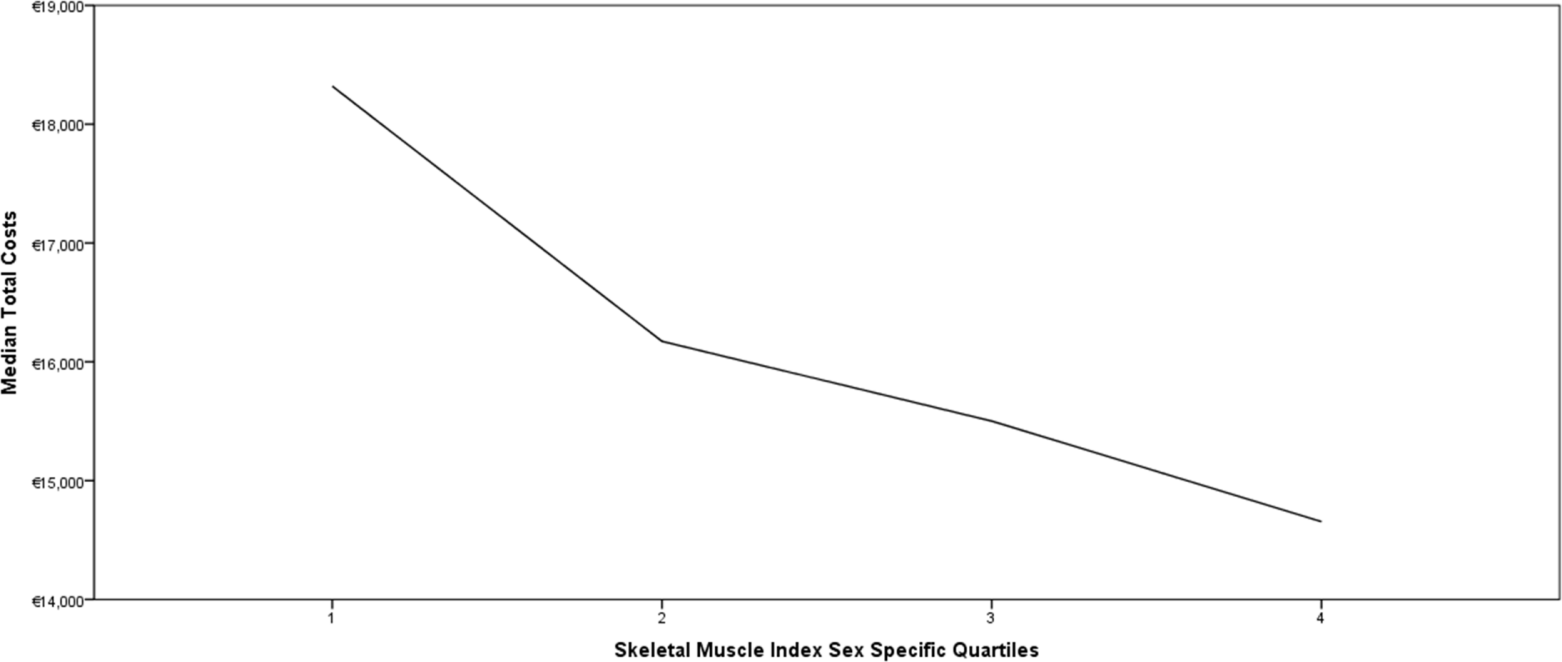
Total hospital costs by skeletal muscle mass in sex-specific quartiles. The total hospital costs significantly decreased per skeletal muscle index sex-specific quartile (*P* = 0.029).

Total costs were higher for patients with low skeletal muscle mass compared with patients with normal skeletal muscle mass (€17,144 [IQR 12,694–25,102] versus €14,961 [IQR 10,744–21,200]; *P*<0.001), resulting in a median difference of €2,183 (12.7%). In univariable linear regression analysis, presence of low skeletal muscle mass was associated with a cost increase of €4,979 (*P* = 0.002). After classifying patients in sex-specific quartiles based on skeletal muscle mass, a decreasing trend in median total hospital costs per patient was observed per incremental quartile (1^st^ quartile €18,320; 2^nd^ quartile €16,172; 3^rd^ quartile €15,501; 4^th^ quartile €14,655; *P* = 0.029, Fig 1).

**Fig 2:**
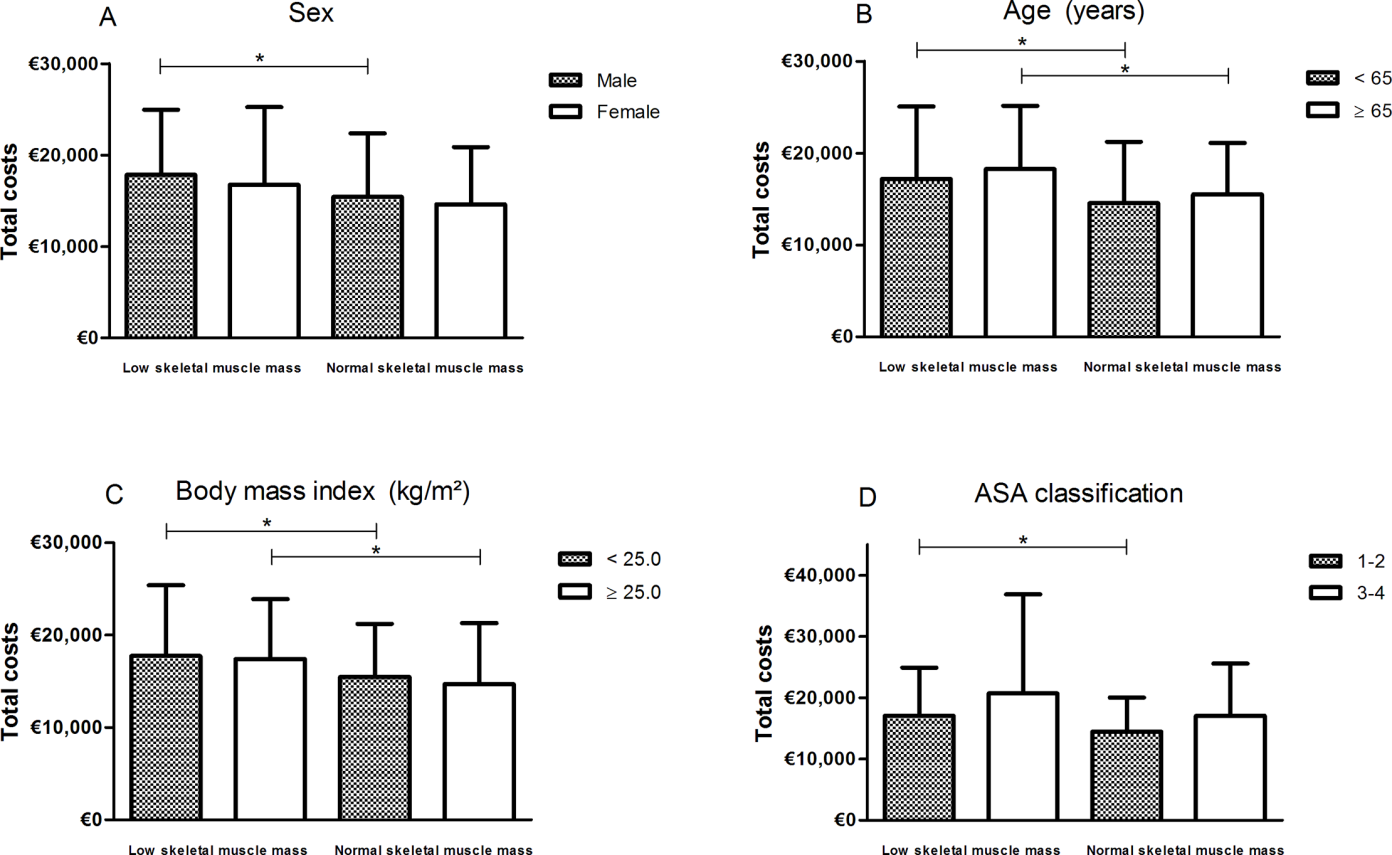
Total hospital costs stratified by the presence of low skeletal muscle mass per patient characteristic. * *P*<0.05.

Median hospital costs were also higher for patients with low skeletal muscle mass compared to patients without low skeletal muscle mass within the subgroup of males (€17,823 [IQR 12,816–24,973] versus €15,444 [IQR 10,363–22,396], *P* = 0.006) and females (€16,755 [IQR 12,081–25,257] versus €14,606 [IQR 11,152–20,898], *P* = 0.121) (Fig 2A), patients <65 years (€17,184 [IQR 12,619–25,073] versus €14,572 [IQR 10,151–21,250], *P* = 0.025) and ≥65 years (€18,256 [IQR 12,808–25,131] versus €15,490 [IQR 11,060–21,098], *P* = 0.041) (Fig 2B) and with overweight (€17,392 [IQR 12,808–23,851] versus €14,667 [IQR 10,277–21,263], *P* = 0.018) and under- or normal weight (€17,410 [IQR 12,457–25,351] versus €15,444 [IQR 10,905–21,179], *P* = 0.048) (Fig 2C).

### The influence of low skeletal muscle mass and postoperative complications on total hospital costs

**Fig 3:**
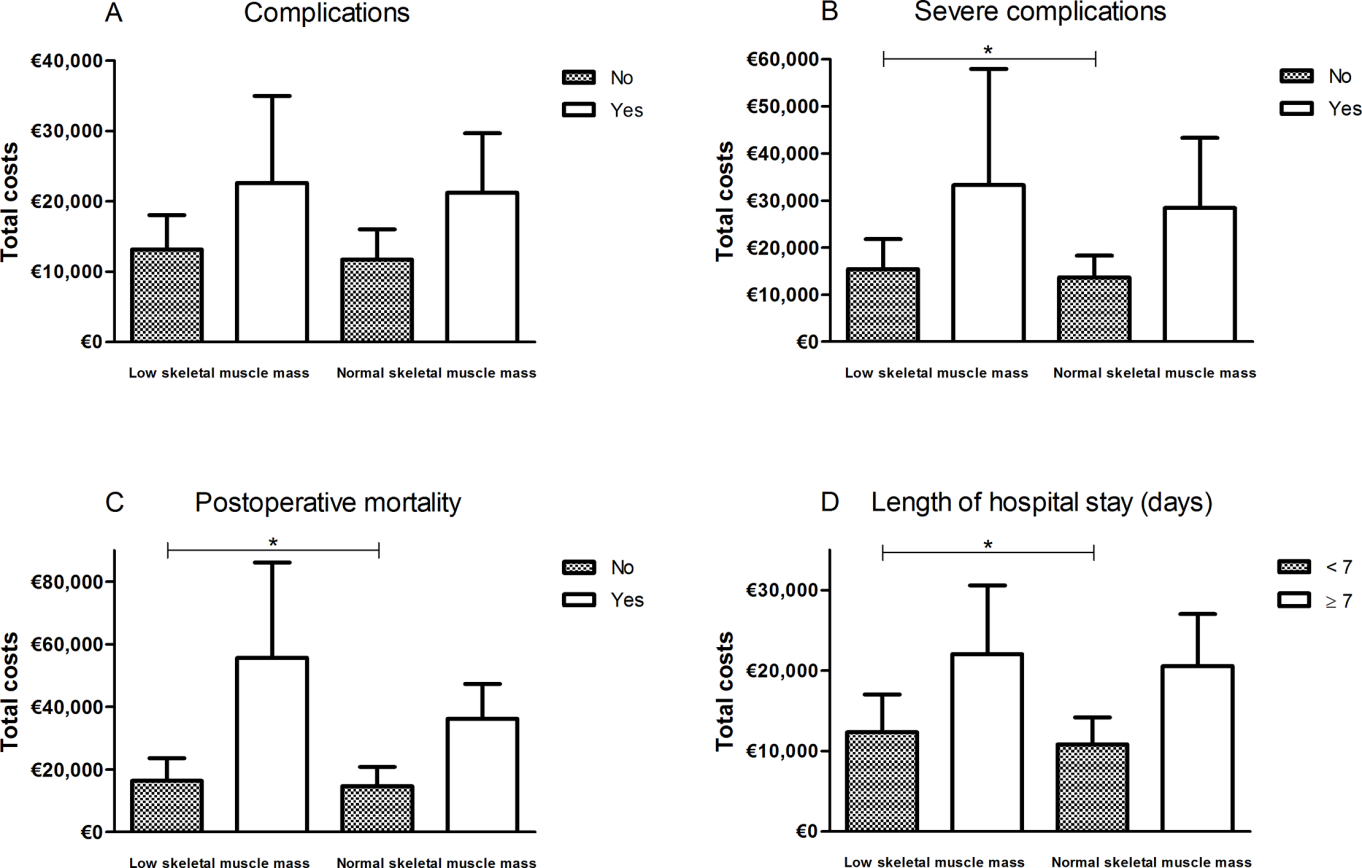
Total hospital costs stratified by the presence of low skeletal muscle mass per treatment outcome. * *P*<0.05.

**Fig 4:**
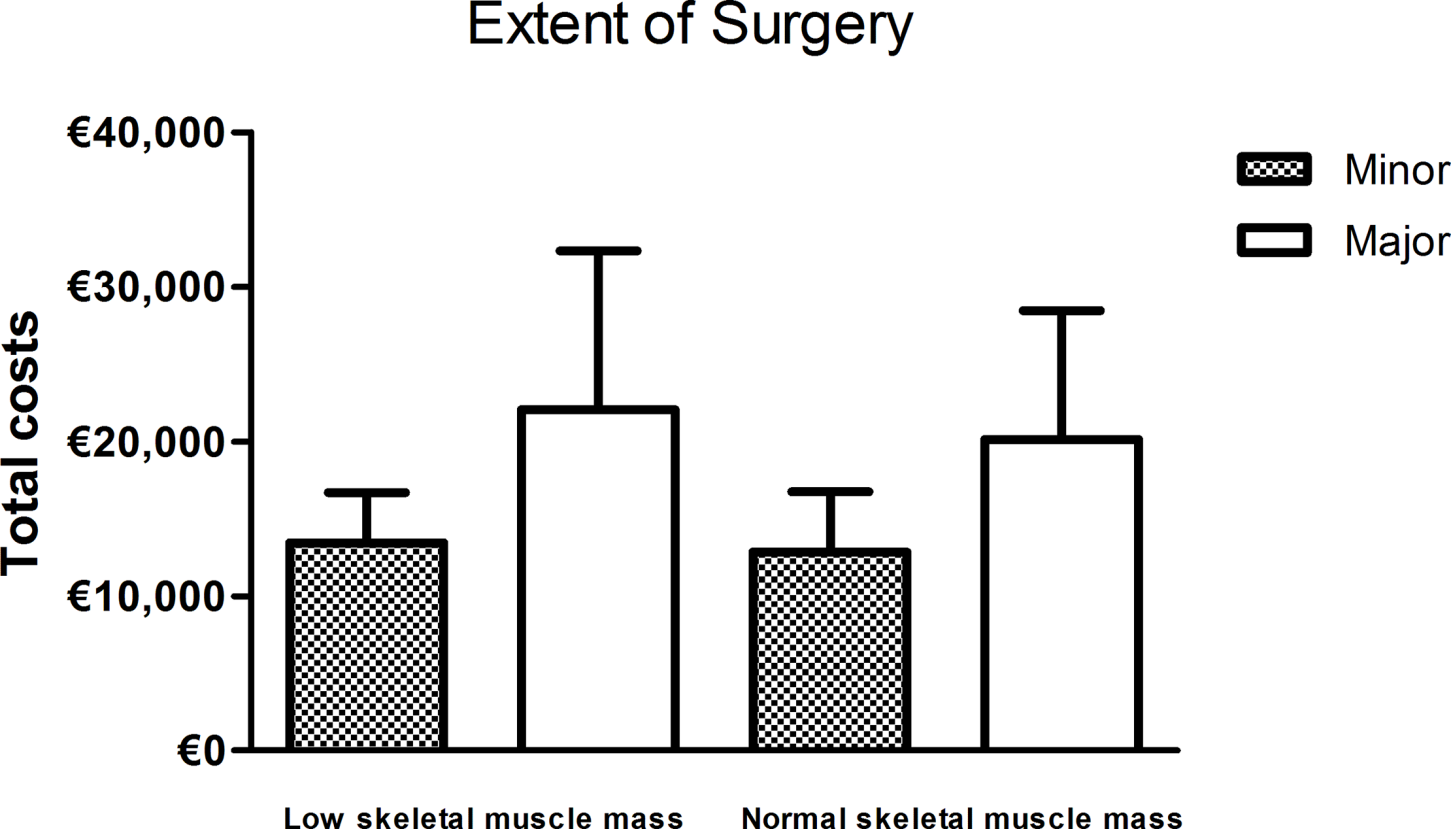
Total hospital costs stratified by the presence of low skeletal muscle mass per surgical treatment group. Major surgery included hepatic resections of at least two segments and a wedge resection or the resection of at least three hepatic segments, pancreatic surgery, and pelvic exenteration for locally advanced rectal cancer. Minor surgery included less than two hepatic segment resections and colorectal resections.

Within the group of patients who did not experience any postoperative complication, the median hospitals costs for patients with low skeletal muscle mass were higher compared with the median costs for patients without low skeletal muscle mass (€13,141 [IQR 9,933–18,046] versus €11,715 [IQR 9,161–16,027], *P* = 0.055), Fig 3A. The same association was observed in patients who did not experience any severe postoperative complications (€15,448 [IQR 12,079–21,789] in patients with low skeletal muscle mass versus €13,658 [IQR 9,938–18,270] in patients without low skeletal muscle mass, *P* = 0.006), Fig 3B. The same effect was observed in patients who did (*P* = 0.084) or did not (*P* = 0.019) die in-hospital or within 30 days postoperatively, and in patients with normal (*P* = 0.041) and prolonged (*P* = 0.434) hospital stay (Fig 3C and 3D). Low skeletal muscle mass was associated with increased total hospital costs in the subgroup of patients undergoing major resections (*P* = 0.099), but not within the subgroups of minor resections (*P* = 0.238), Fig 4.

### Multivariable linear regression analysis

In linear regression analysis, the presence of low skeletal muscle mass was independently associated with higher total hospital costs, after adjusting for the extent of surgery, sex, age, overweight, and ASA classification (Table 4). Presence of low skeletal muscle mass resulted in a cost increase of €4,061 (95% confidence interval [CI] 809–7,312; *P* = 0.015). When skeletal muscle index was used as a continuous parameter, an incremental increase in skeletal muscle index (cm^2^/m^2^) was associated with €278 (95% CI 32–524, *P* = 0.027).

**Table 4:** Multivariable linear regression analysis for the total costs per patient.

	B (Euros)	Standard Error (Euros)	*P*-value
**Low skeletal muscle mass**	4,061	1,654	0.015
**Age (≥65 years)**	484	1,648	0.769
**Sex (male)**	2,126	1,693	0.210
**Overweight (BMI ≥25 kg/m^2^)**	-587	1,650	0.722
**ASA classification (3 or 4)**	7,205	2,044	<0.001
**Extent of surgery (major surgery)**	11,127	1,650	<0.001

Abbreviations: BMI, Body Mass Index; ASA, American Society of Anesthesiologists

## Discussion

In this study in a Western-European healthcare system among patients undergoing gastro-intestinal cancer surgery we found that the costs were €2,183 higher in patients with low skeletal muscle mass. Furthermore, total costs increased gradually across four levels of the skeletal muscle index for both men and women, which is in line with a previous study [7]. Finally, significant differences in costs in patients with low skeletal muscle mass compared with patients without low skeletal muscle mass were observed in patients undergoing major surgery, while this difference was not significant in patients undergoing minor surgery.

Advanced age is associated with both an increased risk of cancer and an increased risk of postoperative complications [25]. With increasing longevity and an increasingly aging population, the number of older cancer patients is steadily growing. About 58% of all cancers and 69% of cancer deaths occur in patients aged 65 years and older [26]. The number of elderly patients undergoing cancer surgery is also rapidly growing. For example, 50% of patients with colorectal cancer are 70 years or older [27]. Therefore, low skeletal muscle mass, resulting from both age-related sarcopenia and disease-related cachexia, may be a highly interesting parameter in an era of an increasingly ageing population.

Besides increased costs, we found significantly more complications and a significantly longer hospital stay in patients with low skeletal muscle mass, which is in line with current literature [1]. Significant differences in hospital costs between patients with and without low skeletal muscle mass were particularly observed in patients who did not experience any (severe) postoperative complications, did not die during admission or within 30 days postoperatively, or did not have a prolonged hospital stay. This finding suggests that increased costs may be directly related to the occurrence of (severe) complications requiring an increased use of resources (e.g., prolonged (ICU) stay, laboratory tests, radiological examinations and radiological or surgical re-interventions), as previously described [28, 29]. This is underlined by the fact that we found a significant difference in patients undergoing major surgery, known for more complex care and relatively high postoperative complication rates, but not in patients undergoing minor surgery. However, based on our results these costs may preoperatively be predicted by CT-based skeletal muscle mass measurements. Skeletal muscle mass measurements are known for their great inter- and intra-observer agreement [30], while ASA classification, for example, is often considered a parameter that is not eligible for cancer patients and subjective with great inter-rater inconsistency [31, 32]. Low skeletal muscle mass may thus be used as a parameter for case-mix corrections to compare treatment outcomes between centres.

To date, all studies performed to assess the association between low skeletal muscle mass and healthcare costs showed a positive association, favouring patients with normal skeletal muscle mass [7–9, 33, 34]. To our knowledge, this study is the first to show that low skeletal muscle mass may be a highly reliable measure to predict impaired outcome and increased hospital costs in surgical cancer patients in Western-European healthcare systems. Since European healthcare costs are increasingly rising and low skeletal muscle mass suggested to be a remediable condition, antagonizing skeletal muscle mass loss in cancer patients may be a powerful target to reduce hospital resource use and costs. Besides prehabilitation programs (i.e. physical exercise therapy combined with nutritional supplementation), promising results have been shown in animal studies [35].Multiple phase II trials are currently being performed [36]. On the other hand, low skeletal muscle mass may be a measure of advanced disease, or even a final common pathway from cancer to death. Low skeletal muscle mass, as a measure of frailty and physical status of patients, may therefore also be added to case-mix correction models.

Some limitations of this study should be acknowledged. A number of costs during hospital admission (i.e. medication and feeding) may not have been recorded and consequently missed in the analyses. In addition, no data was available after discharge from our hospital. It has previously been shown that patients with low skeletal muscle mass experience a delayed recovery and are more likely not to rehabilitate at home [8, 37]. This may have led to an underestimation of the true total costs in patients with low skeletal muscle mass and thus an underestimation of the difference in costs between patients with and without low skeletal muscle mass. However, the total hospital costs are in line with a previous Dutch study investigating costs after major abdominal surgery in an academic centre [28] and the study of Kirk *et al*. which showed that the greatest difference in costs occurs in the first 30 days after surgery [8]. Due to the retrospective design of this study, CT examinations have not been performed in identical scanners. A previous study, however, showed that fat area measurements (based on the same principle) on CT examinations performed with various scanners in individuals had excellent agreements, independent of the scanner used [38]. Some of the non-significant differences found may be explained by a limited sample size. Finally, we only investigated the association between skeletal muscle mass and healthcare costs, rather than sarcopenia, characterized by both skeletal muscle mass and function loss [39], or cachexia, characterized by both skeletal muscle mass and body weight loss [40]. Although low skeletal muscle mass a hallmark parameter of both syndromes, future studies should confirm our results in patients with sarcopenia or cachexia.

In conclusion, total hospital costs are €4,061 higher in patients with low skeletal muscle mass compared with patients with normal skeletal muscle mass after correction for extent of surgery and ASA classification. The search for a treatment of low skeletal muscle mass might therefore lead to a reduction of complications and hospital costs in an era of incremental healthcare costs and an increasingly ageing population. Future prospective studies regarding skeletal muscle mass should consider including total costs as outcome measure.
